# Cesarean Scar Implantation in the Evolution of Placenta Accreta Spectrum: Implications for Recognition and Clinical Management

**DOI:** 10.1097/og9.0000000000000182

**Published:** 2026-07-09

**Authors:** Kathryn Crofton, Sloane York, Laura Laursen

**Affiliations:** Rush University Medical Center, Chicago, Illinois

We read with great interest the article regarding cesarean scar (ectopic) pregnancies by Timor-Tritsch et al^1^ in the February 2026 issue. We especially appreciate the “high-risk-for-placenta accreta-spectrum triangle” schema and the review of previously described classification systems. The systematic approach to screening ultrasound examinations and interpretation of ultrasound images is clear and approachable and demystifies a complex clinical condition that currently suffers from limited clinical guidelines based on low quality of evidence.

Timor-Tritsch et al caution, “Failure to recognize this [cesarean scar-placenta accreta] clinical continuum can lead to unanticipated massive hemorrhage if a low-lying cesarean scar pregnancy is mistaken for an inevitable early pregnancy loss and dilatation and curettage is attempted … .” Although we certainly agree that early identification is crucial for both adequate patient counseling and appropriate treatment planning, we assert that uterine aspiration, performed under ultrasound guidance, is an appropriate treatment option for the management of many cesarean scar ectopic pregnancies.

Altay et al,^2^ Bağlı et al,^3^ and Wu et al^4^ have reported clinical success in their respective case series of cesarean scar ectopic pregnancies managed with uterine aspiration monotherapy under ultrasound guidance. We practice at a tertiary referral center and access hub for patients who need to cross state lines for care, and our practice is similar, adding adjuvant measures such as uterine artery embolization or intrauterine balloon tamponade for more complex and later-gestation cases. This quality of evidence is admittedly low. Although small experimental trials have randomized patients with cesarean scar ectopic pregnancy to various combined modalities of treatment, sometimes including uterine aspiration, uterine aspiration as monotherapy for cesarean scar ectopic pregnancy has been reported only in retrospective, observational studies.

We look forward to research that can inform standardized management guidance based on cesarean scar ectopic pregnancy clinical and ultrasound features to minimize patient burden and maximize patient safety. In particular, we envision that the Timor-Tritsch et al “high-risk-for-placenta accreta-spectrum triangle” will be useful for developing a validated risk stratification tool that can identify patients who are most appropriate for uterine aspiration monotherapy and those requiring more invasive or prolonged treatment options.
